# Surgical treatment and clinical outcome in non-inflammatory atlantoaxial degeneration and retro-odontoid pseudotumor

**DOI:** 10.1016/j.bas.2025.105621

**Published:** 2025-09-29

**Authors:** Raimunde Liang, Bernhard Meyer, Vicki M. Butenschoen

**Affiliations:** Department of Neurosurgery, Technical University Munich, School of Medicine, Munich, Germany

**Keywords:** Atlantoaxial degeneration, Atlantoaxial instability, Cervical spine

## Abstract

**Introduction:**

Patients suffering from atlantoaxial degeneration with spinal cord compression may suffer from burdening neck pain and myelopathy, reducing their mobility and quality of life. Formerly known as a distinct entity in rheumatoid arthritis, atlantoaxial degenerative arthrosis with or without atlantoaxial instability and spinal cord compression may occur without being caused by autoimmune or inflammatory factors.

**Research question:**

This study aims to evaluate the outcome of C1-2 fusion in patients with atlantoaxial degeneration.

**Material and methods:**

We retrospectively assessed patients undergoing C1-2 stabilization for symptoms and radiographical signs of atlantoaxial degeneration with or without a retrodental mass in our neurosurgical department from January 2012 to December 2023. Patients with inflammatory and autoimmune diseases were excluded. Radiological parameters, surgical data, and clinical follow-up data were retrieved from our records to investigate the clinical outcome.

**Results:**

We included 43 patients suffering from refractory neck pain and/or myelopathy for further analysis. The mean age was 73 years. All patients underwent C1-2 fixation and 60.5 % obtained a decompression via C1 laminectomy for myelopathy. Median mJOA score improved significantly from 12/18 preoperatively to 14/18 at a median follow-up of nine months. The mean preoperative atlantodental distance was 2.5 mm. In total, pain relief was achieved in 93.5 % of the patients, and 90.5 % of the patients with preoperative myelopathy improved on the mJOA scale at follow-up.

**Discussion and conclusion:**

C1-2 fusion achieved satisfying results in patients suffering from non-inflammatory atlantoaxial degeneration. Patients suffering from neck pain and symptoms of myelopathy improved until follow-up with the posterior approach.

## Highlights

• C1-2 fusion improves myelopathy and neck pain in atlantoaxial degeneration.
• Degenerative mass does not regress after fixation, unlike inflammatory pannus.
• Additional decompression improves neurological outcomes in selected patients.

## 1 Introduction

Degeneration of the atlantoaxial joint may be associated with atlantoaxial instability, generally characterized by excessive mobility of the two cervical vertebrae forming the atlantoaxial joint: C1 (also referred to as the atlas) and C2 (the axis). It presents the most mobile joint of the spine (Goel, 2021a; Panjabi et al., 1988; Dvorak et al., 1988), and instability may be caused by congenital syndromes such as Down syndrome or skeletal dysplasia in children, inflammatory diseases such as rheumatoid arthritis (RA) (Dvorak et al., 1989), or chronic degeneration of the atlantoaxial joint (Goel, 2021b).

With the optimization of RA treatment, inflammatory diseases with severe atlantoaxial instability become less common, and patients suffering from degenerative atlantoaxial arthrosis with distinct characteristics become more relevant. Patients presenting with atlantoaxial degeneration may suffer from burdening neck pain (Schaeren and Jeanneret, 2005), occipital neuralgia (Ehni and Benner, 1984), and neurological deficits due to spinal canal narrowing with symptoms of myelopathy (Finn et al., 2007), and often go unrecognized (Grob et al., 2006). Due to degeneration, the formation of a retrodental mass similar to inflammatory diseases can still occur, resulting in spinal cord compression. Surgical treatment, such as rigid posterior fixation, may improve symptoms and odontoid mass resolution (Finn et al., 2007). However, while commonly described in patients with inflammatory diseases, reports on patients with atlantoaxial degeneration are lacking. This study, therefore, aims to further investigate the effects of surgical treatment on degenerative atlantoaxial arthrosis.

## 2 Material and Methods

### 2.1 Study design

We performed a single-center retrospective cohort study at the neurosurgery department at a tertiary care hospital in Munich, Germany.

### 2.2 Patient cohort

Patients surgically treated for atlantoaxial degeneration between January 2012 and December 2023 were included. Subjects with a history of rheumatoid arthritis, other inflammatory diseases, and osteomyelitis, as well as patients with traumatic or neoplastic retro-odontoid pannus, were excluded. Symptoms such as pain, motor deficits, and myelopathy which was defined by motor and sensory impairment as well as gait disturbance and/ or dysfunction of bladder and sphincter control and graded by the modified Japanese Orthopedic Association (mJOA) score were assessed before and after surgery and at follow-up examinations. Presence of retro-odontoid was defined as thickness of retro-odontoid soft-tissue >3 mm on midsagittal MRI (Shi et al., 2019). Moreover, the lateral atlantodental interval (ADI) was compared in sagittal images using preoperative Computer Tomograms (CT) scans. An increasing ADI exceeding 3 mm in functional X-ray diagnostics was defined as atlantoaxial macroinstability. Surgical data such as surgery duration, type of fusion, decompression when performed, and surgical complications were recorded.

### 2.3 Statistical analysis

Statistical analyses were performed using GraphPad Prism Version 9.4.1. Categorical data was compared using the chi-square test as needed. Mean values were compared using the independent-samples *t*-test. A non-parametric Kruskal-Wallis test followed by Bonferroni post-hoc test, was used for comparison among several groups for non-normal distributed variables. All tests were performed two-sided. A p-value <0.05 was considered significant.

### 2.4 Ethical considerations

This study was performed by ethical standards outlined in the Declaration of Helsinki. Our local ethics committee approved the study before data collection (registration number 2022-237-S-SR). Due to the retrospective nature of our research, informed consent was waived by our local ethics committee.

## 3 Results

### 3.1 Patient population

We included 43 patients with a mean age of 73 years (range 39–85). Patients were primarily female (58.1 %, n = 25), 41.9 % (n = 18) were male. Patients were explicitly investigated for symptoms of underlying autoimmune disease. None of the patients included had a history of rheumatoid arthritis nor any stiffness of small joints such as finger joints or elevated inflammatory markers (C-reactive protein) or corticosteroid medication. On the contrary, 12 patients underwent C1-2 fusion due to rheumatoid arthritis during this period and were excluded from the present cohort.

### 3.2 Indications for surgical treatment

**Fig. 1:**
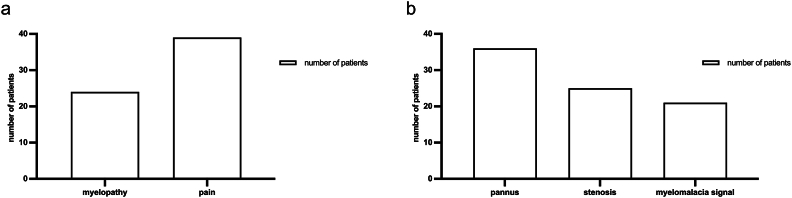
**Distribution of clinical (a) and radiological (b) findings among the patient cohort.** Pain was the main symptom, and a substantial proportion showed signs of cervical myelopathy. Radiologically, retro-odontoid mass formation was evident in 83.7 %, often accompanied by spinal cord compression and myelomalacia.

At initial presentation, 90.7 % of the patients reported severe burdening neck pain (n = 39), which did not respond to non-surgical therapy, and 69.8 % exhibited myelopathic symptoms due to spinal cord compression (n = 30) (Fig. 1). Within this patient group, ten exhibited motor deficits (23.3 %), including tetraparesis (defined as muscle strength graded ≤3 on the Medical Research Council scale in all four extremities) in six patients, hemiparesis in two patients, and two suffering from proximal motor deficits of the upper extremities. Gait disturbance was observed in all myelopathic patients, and fine motor impairment was present in 25 cases (58.2 %). Where documented, myelopathic signs typically evolved over weeks to a few months, whereas neck/suboccipital pain often preceded referral by many months to several years. Duration was inconsistently recorded, precluding quantitative analysis.

All patients underwent dorsal arthrodesis of the C1-2 joint. In case of severe myelopathy and spinal compression on preoperative imaging, additional decompression was generally approached posteriorly via atlas arch resection or repositioning. In certain patients, ventral decompression via transnasal odontoid resection was evaluated based on their clinical presentation and radiological findings. This approach was only considered when posterior decompression was deemed insufficient.

### 3.3 Radiological findings

Bony erosions were diagnosed in 51.2 % (n = 22) on preoperative CT scans. The mean ADI was 2.5 mm (range 0.6–10.5 mm) preoperatively and 2.3 mm (range 0.6–5.6 mm) postoperatively. We found no statistically significant difference between the pre-and postoperative ADI (p = 0.232). Functional X-ray diagnostics was available in 12 patients and revealed atlantoaxial macroinstability in eight patients. Standardized AP open-mouth radiographs were not routinely obtained. Preoperative MRI showed retro-odontoid mass in 36 patients, with severe compression of the spinal cord observed in 25 patients, while a myelomalacia signal was detected in 21 patients on axial and sagittal T2 sequencing (Fig. 1). Degenerative basilar impression was found in three patients, and non-rheumatoid cranial settling was observed in one patient.

### 3.4 Surgical treatment

C1-2 arthrodesis via the Goel-Harms technique (Harms and Melcher, 2001) and additional posterolateral fusion were performed on all 43 patients, no transarticular screws via the Magerl-Grob technique were used (Jeanneret and Magerl, 1992; Grob et al., 1991). In 25 patients with myelopathy and/ or motor deficits and compression of the spinal cord in MRI imaging, we performed decompression via atlas arch resection. An exemplary case is described and illustrated in Fig. 1. In five patients with clinical myelopathy, we opted for no decompression due to a sufficiently wide spinal canal. The mean surgery duration was 125 min (range 64–274). Intraoperative neurophysiological monitoring was not used.

Endoscopic transnasal odontoid resection was performed in six patients (14 %) after a median of seven days (Butenschoen et al., 2020). The reasons were a lack of improvement after the first surgery in three patients with, in one case, transient aggravation of motor deficits immediately after surgery. In the other three patients, resection of the retro-odontoid mass was performed according to the treatment plan due to the presence of severe anterior compression and significant myelopathy on the MRI. In two cases, odontoid resection was evaluated as a secondary step during the outpatient visit; however, it was deemed unnecessary after a satisfactory outcome in clinical follow-up. Histopathological results were available for five of six patients showing degenerative non-inflammatory tissue.

Perioperative complications included three asymptomatic cases of iatrogenic vertebral artery compromise related to C2 screw trajectories; all were asymptomatic and identified on postoperative CT/CTA as partial transverse-foramen breach with the screw abutting the artery. There was no intraoperative hemorrhage, no thromboembolic complication, and no endovascular treatment was required; all patients remained neurologically intact at last follow-up. The overall complication rate was 20.9 %, including urinary tract infections (n = 2), wound healing disorders requiring antibiotic treatment (n = 3), pulmonary embolism (n = 1), and the three vertebral artery injuries.

### 3.5 Clinical outcome

Overall, 78 % of patients improved in their main clinical symptoms, two patients deteriorated (4.9 %), and 17.1 % reported a stable clinical status (n = 7) directly after surgery. The median score on the mJOA scale remained stable from 12/18 preoperatively to 12/18 directly after surgery before discharge. It improved to a median score of 14/18 in a follow-up assessment available for 21 patients (p = 0.008) after a mean of nine months (range one month to five years).

More specifically, 90.5 % of our patients with available follow-up mJOA data and preoperative myelopathy (19/21) improved at least one point on the mJOA scale, and two remained stable. The improvement score was two points. Of 31 patients with available follow-up information, 29 (93.5 %) reported significant relief of burdening neck pain. Two patients had postoperative symptom alleviation but presented with recurrent pain due to screw loosening.

The clinical outcome, in terms of symptom improvement or deterioration, was not significantly associated with age (p = 0.811) or ADI (p = 0.37).

Of all patients who underwent decompression for symptoms of myelopathy, 84 % showed improved myelopathy symptoms. Meanwhile, in patients with myelopathy who underwent fixation without decompression, 40 % showed improvement, while 60 % remained stable. This trended towards significance but failed to reach it, possibly due to the small number of patients included (p = 0.065). Notably, although none of the patients without additional decompression exhibited severe stenosis, two showed a myelomalacia signal on MRI imaging.

In comparison, those patients requiring additional transnasal odontoid resection demonstrated more severe baseline pathology and higher morbidity, as summarized in Table 1.

**Table 1:** Comparison of clinical, radiological, and outcome parameters in patients with and without ventral surgery.

Variable	C1-2 fixation without ventral surgery (n = 37)	Additional transnasal odontoid resection (n = 6)
**Demographics**		
Age, years — mean	72	76
Male/Female	13/24	5/1
**Clinical presentation**		
Neck/suboccipital pain — n (%)	35 (95)	4 (67)
Myelopathy — n (%)	24 (65)	6 (100)
mJOA score (preop) — median	12	11
Focal motor deficit — n	7	3
**Imaging**		
Retro-odontoid mass — n (%); median size	29 (78); 6 (range 1–12)	6 (100); 8,5 (range 4–12)
T2 myelomalacia — n (%)	15 (41)	6 (100)
**Outcome**		
mJOA score (at discharge) — median	13	11
mJOA score (at follow up) — median	16 (of 15 available patients)	14 (all patients available)
**Complication rate**		
Surgical complications — n (%)	5 (13.5)	1 (16.7)
Overall complications — n (%)	7 (19)	2 (33)
Length of stay, days — median	7	22.5

**Fig. 2:**
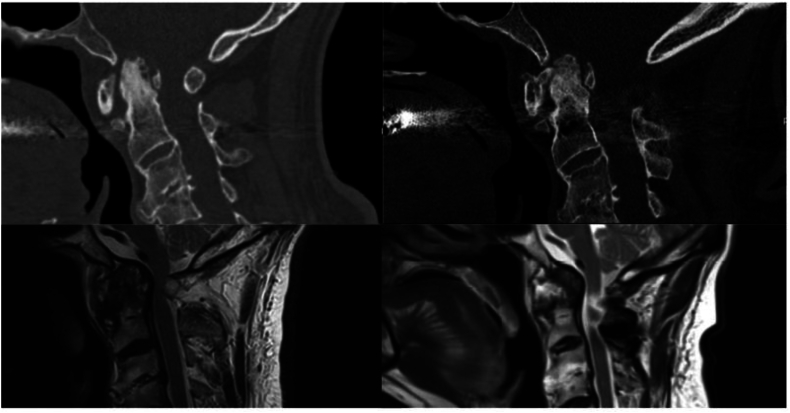
**Pre- and postoperative imaging of a patient undergoing C1-2 stabilization and decompression surgery.** This figure presents a representative case of a 62-year-old male with a history of neck pain lasting several years, clinical examination revealed hyperreflexia and marked unsteadiness on tandem walking. Preoperative imaging identified a retrodental mass leading to significant spinal stenosis. The patient underwent C1-2 stabilization, atlas arch resection, and posterolateral fusion. The patient exhibited symptom improvement at the one year follow-up, although the retrodental mass remained unchanged on imaging.

**Fig. 3:**
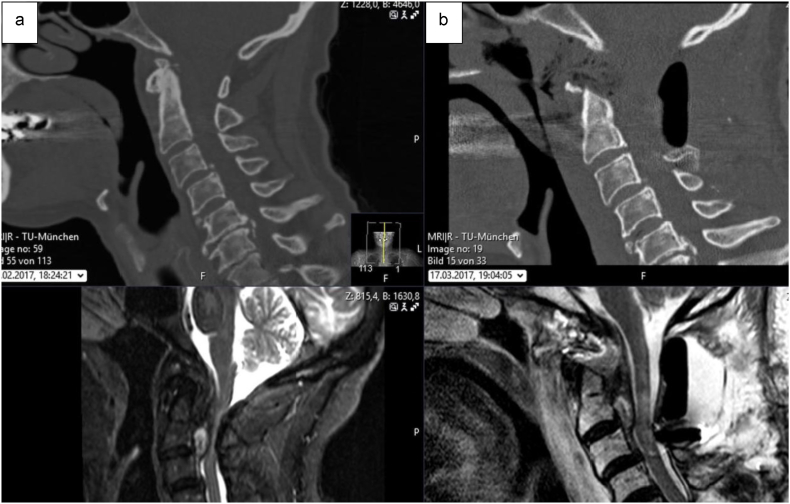
**Pre- and postoperative imaging of a patient treated with C1–2 stabilization and decompression and subsequent transnasal endoscopic odontoid tip resection.** The patient presented with gait instability and impaired fine motor skills in both hands. Imaging revealed severe spinal stenosis caused by a significant ventral retrodental mass (Fig. 3a). A two-stage surgical plan was developed. First, dorsal stabilization of C1-2, resection of the atlas arch, and posterolateral fusion was performed, resulting in adequate dorsal decompression, though significant ventral compression remained. This was followed by transnasal endoscopic resection of the odontoid tip and decompression (Fig. 3b), which reduced the spinal stenosis caused by the odontoid mass. At the two-month follow-up, the patient showed symptom improvement.

Notably, retro-odontoid masses were observed in all patients treated with odontoid resection, with a larger median size (8.5 mm) compared to the C1-2 fixation-only group (6 mm, range 1–12). Functional status, measured by the mJOA score, was slightly worse at baseline in the dens resection cohort (median 11 vs. 12) and remained lower both at discharge (11 vs. 13) and at follow-up (14 vs. 16). The length of hospital stay was substantially longer in patients undergoing additional odontoid resection (median 22.5 vs. 7 days). Illustrative cases are shown in Fig. 2, Fig. 3.

Follow-up imaging, consisting of either MRI or CT scans, was available for nearly half of the patients in our cohort (46.5 %). Despite dorsal fixation, the imaging did not reveal any evidence of regression of the retrodental mass, suggesting that the intervention may not have impacted the mass as expected.

## 4 Discussion

In this study, surgical treatment with C1-2 stabilization in patients with atlantoaxial degeneration achieved symptom improvement in most cases. Additional posterior decompression appeared particularly effective compared to C1-2 stabilization alone, in particular in patients suffering from myelopathy.

To date, scarce data is available regarding the presentation and treatment of patients with atlantoaxial degenerative arthrosis and retro-odontoid mass compared to rheumatoid arthritis. Our case series with 43 patients is among the largest investigating C1-2 fixation in this context.

In rheumatoid patients, inflammatory processes leading to erosive changes in the atlantoaxial joint are assumed to result in atlantoaxial instability. Retro-odontoid pannus typically tends to regress following atlantoaxial fixation in rheumatoid patients. Recent studies suggest that posterior C1-2 fixation and/or posterior decompression (Janssen et al., 2020) often eliminate the need for anterior resection of the pannus (Goel et al., 2021; Shah et al., 2016), which is a procedure associated with significant complication risks (Yeh et al., 2019; Goldschlager et al., 2015). In cases of irreducible retro-odontoid mass, anterior resection and decompression are recommended, as described by Janssen et al. (2020).

Similarly, this was also described in atlantoaxial degeneration (Takahata et al., 2023; Barbagallo et al., 2013; Niwa et al., 2021). Our study observed notable differences in our cohort compared to inflammatory atlantoaxial instability. While inflammatory atlantoaxial instability is typically characterized by an ADI >3 mm (Janssen et al., 2020; Gillick et al., 2015; Joaquim et al., 2015), our findings indicated a mean preoperative ADI of only 2.5 mm. Measurements were performed on preoperative CT scans as preoperative flexion/ extension X-rays and AP open-mouth imaging were only available in a limited subset of patients rendering further analyses infeasible. Nevertheless, Takahata et al. (2023) reported that patients with non-rheumatic retro-odontoid pseudotumor exhibited a mean ADI of 3 mm, attributed to an overall narrowing of ADI with age. Being a frequent occurrence in rheumatoid arthritis (Janssen et al., 2020; Joaquim et al., 2015; Joaquim and Appenzeller, 2014), our case series identified only one patient with cranial settling and three with basilar impressions. Other advanced stages of atlantoaxial instability were not observed. We hypothesize that, unlike in rheumatoid arthritis, patients with non-inflammatory atlantoaxial degeneration exhibit less C1-C2 instability.

Our study suggests a trend in favor of additional posterior decompression in case of myelopathic symptoms. This finding aligns with the proposed underlying pathology, indicating that non-inflammatory mass formation, unlike rheumatic pannus with hypermobility, does not typically regress spontaneously following stabilization, necessitating additional posterior or ventral decompression or repositioning. This hypothesis is supported by the observation that the retrodental mass did not regress at follow-up imaging in our study cohort. Furthermore, our results suggest that myelopathy in this context may not solely result from stenosis but also from instability itself, further supporting the need for decompression even in the absence of radiologically evident stenosis. Although representing only a small subset, histology in the five available cases (ventral-decompression subgroup) showed no relevant inflammatory activity, consistent with a degenerative phenotype. Clinically, recognition is often delayed: C1–C2 osteoarthritis is very painful yet frequently misdiagnosed, and patients commonly present first with non-specific neck pain, with myelopathic signs emerging only later. This likely contributes to the high proportions of clinical signs of myelopathy of nearly 70 % as well as myelomalacia in the imaging of nearly 50 % observed in our cohort. We therefore recommend vigilance for such non-specific presentations, a careful functional examination of the cervical spine, and incorporation of standardized AP open-mouth (odontoid/C1–2; transoral/transbuccal) radiographs together with lateral flexion–extension radiographs into the diagnostic work-up. In addition, routine histopathological sampling whenever retro-odontoid tissue is resected should be performed to distinguish etiologies. Further odontoid resection was performed in a select subset of patients. While odontoid resection is typically considered several weeks after C1-2 stabilization in rheumatoid patients due to potential spontaneous resolution (Janssen et al., 2020), we opted for an early endoscopic transnasal odontoid resection in patients with inadequate or ongoing compression following the initial surgery based on the assumption that degenerative mass is likely to persist. The pathology indicates an earlier and broader requirement for ventral access to achieve optimal clinical outcomes. While the transoral approach was historically considered the gold standard for ventral decompression of the odontoid process, it is associated with complications such as dysphagia and postoperative airway obstruction, which may necessitate tracheostomy (Gillick et al., 2015). Consequently, the less invasive endoscopic transnasal approach was selected for its minimally invasive nature, linked to earlier extubation and lower risk of velopharyngeal incompetence (Butenschoen et al., 2020).

All in all, improvement of clinical symptoms after C1-2 stabilization was found in more than 90 % of our patients, aligning with reports on inflammatory diseases and smaller case series on atlantoaxial degeneration (Goel et al., 2021; Takahata et al., 2023; Bydon et al., 2015; Kleinstuck et al., 2021; Fung et al., 2018). Interestingly, we observed a higher occurrence of atlantoaxial degeneration in females, while a study by Niwa et al. (2021) found a male predominance.

The study's limitations primarily stem from its retrospective design, which may introduce inherent data collection such as lacking validated pain and outcome assessment tools and comprehensive diagnostics for inflammatory diseases as well as and analysis biases. Additionally, the relatively small sample size reflects the possibly low prevalence of atlantoaxial degeneration, which is often underreported and may result in fewer patients being referred to specialized spine centers for evaluation and potential surgical intervention. Due to the limited cohort size, further stratification of subgroups, such as those based on the presence of myelopathy or retro-odontoid mass, was not feasible. These patient subsets may exhibit distinct pathophysiological mechanisms, clinical outcomes, and treatment considerations. Specifically, the limited sample size affects the generalizability of the significance of additional decompression. Moreover, the median follow-up time of 15.5 months might not fully represent the final outcome, as the time described in the literature for mass reduction can take up to 40 months (Barbagallo et al., 2013). Imaging follow-up was available in a subset of patients and was not obtained according to a standardized protocol. Scans were primarily performed for clinical reasons and rarely extended into late time windows in which regression of retro-odontoid tissue has been reported. Accordingly, our data do not allow a formal analysis of mass regression after posterior fixation. Prospective studies with protocolized MRI at predefined intervals (e.g., 12, 24, and 36 months) are needed.

The lack of laboratory investigations, such as rheumatoid factor or HLA typing, to definitively exclude inflammatory arthropathy represents a notable limitation of the study. Additional inflammatory diseases, such as calcium pyrophosphate dihydrate deposition (CPPD), were not investigated - presenting a limitation even if cases with retro-odontoid pannus are rare (Klineberg et al., 2014).

## 5 Conclusion

This retrospective cohort study provides insights into the surgical management and outcomes of atlantoaxial degeneration. Fixation of the C1-C2 joint, particularly in addition to decompression surgeries, effectively alleviated patient symptoms.

## Statement of ethics and consent to participate

The presented study meets the ethical standards outlined in the Declaration of Helsinki. Ethics approval was obtained by our local ethics committee, and the favorable vote was registered under the number 2022-237-S-SR.

## Availability of data and materials

The datasets used and analyzed during the current study are available from the corresponding author upon reasonable request.

## Contribution

Conception and design: BM.

Acquisition of data: VB, RL.

Analysis and interpretation of data: VB.

Manuscript draft: VB, RL.

Critical revision for important intellectual content: BM.

Final approval: VB, RL, BM.

## Funding

The 10.13039/501100005713Technical University of Munich funded the study.

## Conflict of interest

We, at this moment, declare no conflict of interest.

## References

[bib1] Barbagallo G.M., Certo F., Visocchi M., Palmucci S., Sciacca G., Albanese V. (2013). Disappearance of degenerative, non-inflammatory, retro-odontoid pseudotumor following posterior C1-C2 fixation: case series and review of the literature. Eur. Spine J..

[bib2] Butenschoen V.M., Wostrack M., Meyer B., Gempt J. (2020). Endoscopic transnasal odontoidectomy for ventral decompression of the craniovertebral junction: surgical technique and clinical outcome in a case series of 19 patients. Oper. Neurosurg..

[bib3] Bydon M., Macki M., Qadi M., De la Garza-Ramos R., Kosztowski T.A., Sciubba D.M., Wolinsky J.P., Witham T.F., Gokaslan Z.L., Bydon A. (2015). Regression of an atlantoaxial rheumatoid pannus following posterior instrumented fusion. Clin. Neurol. Neurosurg..

[bib4] Dvorak J., Schneider E., Saldinger P., Rahn B. (1988). Biomechanics of the craniocervical region: the alar and transverse ligaments. J. Orthop. Res..

[bib5] Dvorak J., Grob D., Baumgartner H., Gschwend N., Grauer W., Larsson S. (1989). Functional evaluation of the spinal cord by magnetic resonance imaging in patients with rheumatoid arthritis and instability of upper cervical spine. Spine.

[bib6] Ehni G., Benner B. (1984). Occipital neuralgia and the C1-2 arthrosis syndrome. J. Neurosurg..

[bib7] Finn M., Fassett D.R., Apfelbaum R.I. (2007). Surgical treatment of nonrheumatoid atlantoaxial degenerative arthritis producing pain and myelopathy. Spine.

[bib8] Fung M., Frydenberg E., Barnsley L., Chaganti J., Steel T. (2018). Clinical and radiological outcomes of image guided posterior C1-C2 fixation for atlantoaxial osteoarthritis (AAOA). J. Spine Surg..

[bib9] Gillick J.L., Wainwright J., Das K. (2015). Rheumatoid arthritis and the cervical spine: a review on the role of surgery. Internet J. Rheumatol..

[bib10] Goel A. (2021). Indicators of atlantoaxial instability. J. Craniovertebral Junction Spine.

[bib11] Goel A. (2021). Degenerative arthritis of the craniovertebral junction. J. Craniovertebral Junction Spine.

[bib12] Goel A., Darji H., Shah A., Prasad A., Hawaldar A. (2021). Retro-odontoid and retro-C2 body pseudotumor, pannus, and/or cyst. A study based on analysis of 63 cases. World Neurosurg..

[bib13] Goldschlager T., Hartl R., Greenfield J.P., Anand V.K., Schwartz T.H. (2015). The endoscopic endonasal approach to the odontoid and its impact on early extubation and feeding. J. Neurosurg..

[bib14] Grob D., Jeanneret B., Aebi M., Markwalder T.M. (1991). Atlanto-axial fusion with transarticular screw fixation. J. Bone Joint Surg. Br..

[bib15] Grob D., Bremerich F.H., Dvorak J., Mannion A.F. (2006). Transarticular screw fixation for osteoarthritis of the atlanto axial segment. Eur. Spine J..

[bib16] Harms J., Melcher R.P. (2001). Posterior C1-C2 fusion with polyaxial screw and rod fixation. Spine.

[bib17] Janssen I., Nouri A., Tessitore E., Meyer B. (2020). Cervical myelopathy in patients suffering from rheumatoid arthritis-A case series of 9 patients and A review of the literature. J. Clin. Med..

[bib18] Jeanneret B., Magerl F. (1992). Primary posterior fusion C1/2 in odontoid fractures: indications, technique, and results of transarticular screw fixation. J. Spinal Disord..

[bib19] Joaquim A.F., Appenzeller S. (2014). Cervical spine involvement in rheumatoid arthritis--a systematic review. Autoimmun. Rev..

[bib20] Joaquim A.F., Ghizoni E., Tedeschi H., Appenzeller S., Riew K.D. (2015). Radiological evaluation of cervical spine involvement in rheumatoid arthritis. Neurosurg. Focus.

[bib21] Kleinstuck F.S., Fekete T.F., Loibl M., Jeszenszky D., Haschtmann D., Porchet F., Mannion A.F. (2021). Patient-rated outcome after atlantoaxial (C1-C2) fusion: more than a decade of evaluation of 2-year outcomes in 126 patients. Eur. Spine J..

[bib22] Klineberg E., Bui T., Schlenk R., Lieberman I. (2014). Retro-odontoid calcium pyrophosphate dehydrate deposition: surgical management and review of the literature. Evid. Base Spine Care J..

[bib23] Niwa R., Takai K., Taniguchi M. (2021). Nonrheumatoid retro-odontoid pseudotumors: characteristics, surgical outcomes, and time-dependent regression after posterior fixation. Neurospine.

[bib24] Panjabi M., Dvorak J., Duranceau J., Yamamoto I., Gerber M., Rauschning W., Bueff H.U. (1988). Three-dimensional movements of the upper cervical spine. Spine.

[bib25] Schaeren S., Jeanneret B. (2005). Atlantoaxial osteoarthritis: case series and review of the literature. Eur. Spine J..

[bib26] Shah A., Jain S., Kaswa A., Goel A. (2016). Immediate postoperative disappearance of retro-odontoid "pseudotumor. World Neurosurg..

[bib27] Shi J., Ermann J., Weissman B.N., Smith S.E., Mandell J.C. (2019). Thinking beyond pannus: a review of retro-odontoid pseudotumor due to rheumatoid and non-rheumatoid etiologies. Skelet. Radiol..

[bib28] Takahata M., Hyakkan R., Oshima S., Oda I., Kanayama M., Hyakumachi T., Fujita R., Endo T., Kajino T., Iwasaki N. (2023). Cervical myelopathy caused by non-rheumatic retro-odontoid pseudotumor: an investigation of underlying mechanisms and optimal surgical strategy. Glob. Spine J..

[bib29] Yeh M.Y., Huang W.C., Wu J.C., Kuo C.H., Chang H.K., Tu T.H., Chang P.Y., Yen Y.S., Cheng H. (2019). Suture repair in endoscopic surgery for craniovertebral junction. Neurospine.

